# Could it be colic? Horse-owner decision making and practices in response to equine colic

**DOI:** 10.1186/1746-6148-10-S1-S1

**Published:** 2014-07-07

**Authors:** Claire E Scantlebury, Elizabeth Perkins, Gina L Pinchbeck, Debra C Archer, Robert M Christley

**Affiliations:** 1 Department of Epidemiology and Population Health, Institute of Infection and Global Health, University of Liverpool, Leahurst Campus, CH64 7TE, United Kingdom; 2 Health Services Research Department, Institute of Psychology Health and Society, University of Liverpool, Liverpool, L69 3GL, United Kingdom

**Keywords:** equine colic, horse-owners, sociology, mixed methods research, epidemiology, grounded theory, qualitative data

## Abstract

**Background:**

Little is known about lay understanding and decision making in response to colic. Horse-owners/carers are key to identifying colic and initiating veterinary intervention. Understanding how owners think and act in relation to colic could assist veterinary surgeons in tailoring information about colic with the aim of improving colic outcomes.

**Methods:**

A mixed methods approach was employed including qualitative in-depth interviews and a cross-sectional questionnaire. Qualitative data were analysed using Grounded theory to conceptualise processes involved in horse-owner management of colic. Following this, a cross-sectional survey was designed to test these concepts. Cluster analysis explored the role of the human-horse relationship upon colic management strategies.

**Results:**

Fifteen horse-owners with a range of colic experience participated in the interviews. A theoretical conceptual model was developed and described how horse-owners’ recognised, assessed and responded to colic. Three main management strategies were used including ‘wait and see’, ‘lay treatments’ and ‘seek veterinary assistance’. Actions in response to colic were moderated by owners’ experience of colic and interpretation of the severity of colic signs. A postal questionnaire gathered data from 673 horse-owners from the North-West of the UK. The majority (605, 89.9%) of respondents were female. Cluster analysis revealed 5 meaningful groups of horse-owners based upon assessment of questionnaire items on the human-horse relationship. These groups included 2 professional and 3 amateur owner typologies. There were differences in the responses to some questionnaire items among the identified groups.

**Conclusions:**

This study describes lay understanding and management of colic among a population of horse-owners from the North-West of the UK. The information may serve as a basis upon which to tailor existing programmes designed to educate owners about colic management strategies, and may inform veterinarians’ interactions with horse-owners.

## Background

Colic is a leading cause of mortality among horses [1,2] and has been estimated to cost the US equine industry $115.3 million per year [3]. Owners rank colic as a high priority equine health concern [4] and it is a frequent reason for veterinary attendance [5].

Colic may resolve spontaneously or in response to medication, but some forms may result in severe physiological compromise leading to rapid death. In such cases, early recognition of colic by horse-owners and subsequent timely veterinary attendance is essential to increase the chance of a successful outcome [6]. Additionally, veterinary attendance can alleviate pain with prompt treatment; therefore, it is important that horse-owners/primary-carers are aware of colic signs as they play a critical role in initiating veterinary intervention. Despite this, there is little information on how horse-owners’ assess and manage colic episodes. Previous research exploring the pathophysiology and epidemiology of colic has provided much valuable information to assist in the design of colic prevention strategies. However, insight into the sociology of colic management is needed and could further support the design and practical implementation of such strategies.

Previously, only a handful of studies have used sociological methods to study human attitudes and influences on equine management and health [7-11]. This study adopted a mixed-method approach and aimed to identify horse-owners’ beliefs and decisions when faced with an episode of colic.

## Methods

Data collection comprised two phases; 1) in-depth face-to-face interviews with horse-owners and 2) a cross-sectional survey of horse-owners in the North-West of the UK. Analysis of the interviews informed the design of the questionnaire, which aimed to examine the generalisability of the theoretical concepts generated from the qualitative research. Ethical approval was granted by the University of Liverpool Ethics committee prior to study commencement.

### Sample Selection

Horse-owners for both phases of the study were selected from a common sampling frame devised as follows:

1. Randomly selected horse-owners who returned postcards for a previous study (n=838 horses age ≥15 years, 656 horses <15 years) [12].

2. Owners of horses that were discharged post colic surgery from the Philip Leverhulme Equine Hospital (University of Liverpool) (Jan 2008 to September 2010) (n=311).

3. Owners of horses treated for medical colic by the University of Liverpool’s first opinion practice (Jan 2007 to September 2010) (n=109).

4. Horse-owners from a previous recurrent colic study (n=10) [13].

All horse-owners were categorised by their colic experience; recurrent colic, medical colic, surgical colic and no colic experience^1^. Owners’ with incomplete addresses or whose horse had died were excluded from the sampling frame. In total, 1,841 horse-owners were identified.

### Qualitative study

Twenty owners were selected from each category (all 10 from category 4) of colic experience and were sent letters inviting them to participate, followed by a telephone call. Owners were purposefully selected based upon colic experience and to include a range of amateur and professional horse-owners.

The qualitative face-to-face interviews were conducted at a location selected by the participant, recorded with a digital Dictaphone and transcribed *verbatim*. Interviews were semi-structured and included the following topics; the owners’ definition of colic, signs associated with colic, knowledge of colic and owner approaches to colic management. Data were analysed using a Grounded theory approach [14,15]. This involved an iterative process of data coding and revisiting earlier transcripts for comparison as analysis progressed. Initial open codes were developed from line-by-line analysis and captured concepts using labels. As coding progressed, similarities and differences in the codes and the data were identified, resulting in the development of key categories. Axial coding was facilitated by drawing diagrams which helped to order the relationships between the codes and between transcripts. Data saturation was achieved when no new coded themes were emerging from the data (i.e. no new hierarchical concepts) [15].

### Questionnaire study

The qualitative study developed a theoretical model for the management of colic. This was used as a framework for the questionnaire which included the following sections; the human-horse relationship, owner management of a colic episode, owner recognition and assessment of putative colic signs, decision making prompting owners to seek veterinary assistance, and consent to surgery. Questions were designed based upon findings from the interview data along with published and expert knowledge of colic. The human-horse relationship was defined via questions about the owners’ view of the horse’s role in their life and their classification of involvement in equestrian activities. This was based, in part, upon Jones’ [16] classification of ‘achievers’, ‘relators’ and ‘riding is a sport’ (see additional file 1). The questionnaire was designed using Teleform software allowing automated entry of data into a database (Access 2007).

The questionnaire was piloted at 2 livery yards among 20 horse-owners and amendments made in response to their feedback. Following this, 1000 addresses were randomly selected from the entire sample.

Questionnaire data were analysed in Minitab, (Minitab Inc, State College, PA) and using ‘R’ (http://cran.r-project.org). In order to explore owner typologies, cluster analysis based on Euclidian distance and Ward’s agglomeration method was performed on the human-horse relationship data. Clusters representing owner typology groups were identified and used to compare questionnaire responses regarding colic management. Chi-square statistics were used to test these comparisons.

## Results

### Qualitative study

A total of 15 interviews were undertaken with a range of horse-owners: 13 females and 2 males (age range 25 to 79); four ‘professional’ owners (i.e. equine activities comprised their primary income) and 11 ‘amateur’ horse-owners. The interviewees varied in their colic experience (see additional file 2).

**Figure 1:**
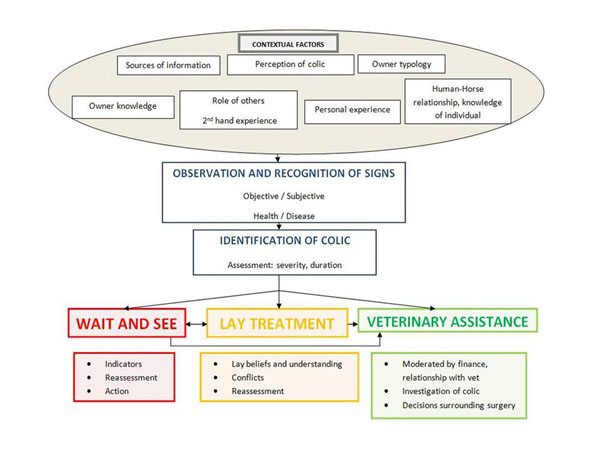
Theoretical diagram: Model of horse-owners’ approaches to management of a colic episode

The theoretical diagram (Figure 1) illustrates the complexity of horse-owner decision-making in response to an episode of colic. The diagram identifies the key elements involved and the relationship between these factors.

### Observation and recognition of signs of colic

Owners described two types of behaviour associated with colic. Firstly, objective, observable behaviours including rolling, stamping, lying down, not eating, box walking, pawing at the ground, sweating, kicking, getting up and down and ears held back. Secondly, subjective signs based on knowledge of the horse and its normal way of being these included, ‘appearing a bit listless’, ‘uneasiness’, ‘not being happy’, ‘being uncomfortable’ and ‘feeling sorry for themselves’. A combination of objective and subjective cues was often reported.

Deviations from expected normal patterns of behaviour alerted owners to a problem which, depending on their knowledge and experience, could be viewed as colic. However, the threshold at which an owner considered there to be a significant change in their horse’s health differed between individuals. The confidence with which owners’ labelled any particular group of signs as colic varied with individual knowledge and experience.

### Identification of colic

Defining a particular set of signs as colic involved; pattern recognition, assessment of signs and perceived severity of signs. Pattern recognition played a role particularly if owners were familiar with colic.

*”I’ve seen cases where there has been a rhythm you know I think both major incidences I’ve experienced, they’ve both been spasmodic in the sense […], you know it came and it went, it eased and it went.”* - Competing amateur 2.^2^

### Assessment of putative colic signs

Assessment would be made of how frequently the observed behaviours were occurring, the nature of faecal output, the perceived severity of signs, how many changes were present, and in some cases, an assessment of gut sounds. In general, colic was understood as a malfunction of the horse’s gut. This was often described as, ‘blockages’ and ‘twists’, both of which were acknowledged to cause pain.

*” his system wasn’t […] digesting properly he’d get these really bad blockages in his bowel.“* - Professional livery yard manager.

‘Twists’ were viewed as serious events and feared due to an association with surgery and risk to the horse.

*”I don’t know whether it was something that happens because their gut is so full, and it makes it twist out of shape or whether […] writhing and carrying on caused some problems [...] but she ended up with a twisted gut, and had to be put down.”* - Amateur/hobbyist 4.

### Interpretation of the severity of colic signs

The intensity, frequency and number of observed behavioural signs led the owner to categorise the colic as mild or severe. This categorisation was an important factor in the decision to call the veterinary surgeon.

Mild colic episodes were difficult for owners to define and were often interpreted as transient discomfort. Owners expressed less concern about mild colic and, unless it became more severe, might not call out a veterinary surgeon.

*”They weren’t bad enough, he wasn’t rolling and he was just that sort of uncomfortable but not bad enough, I mean it didn’t last very long...he could get up and walk around and he’d eat a bit more then he’d go and lie down again [...] they were minor episodes really of wind.”* - Amateur pet/companion.

Signs associated with severe colic included; rolling on the floor, being unable to get up and sweating. In the following example the decision to call the veterinary surgeon was made on the basis of all observed signs interpreted as severe colic.

*” That was really, the worst kind of colic that I have ever seen. She couldn’t lift her head up, […] I came in the morning and she was down on the floor and she was sweating. And I tried to get her up, she wasn’t having that, so I got the vet. Eventually she did stand up but she was shaking, really bad on her feet, and then she would just keep groaning and trying to get down onto the floor again.”* - Amateur/hobbyist 3.

### Stages of colic

The distinction between mild and severe colic was defined by some owners as different ‘stages’ representing increasing severity of colic. In these cases, behavioural signs assisted the recognition of colic and decision to call the veterinary surgeon.

*” The first stage of colic is just slightly...they go down... but then they get straight back up... […] then the second stage of colic is where they are on the floor more times than they are on their feet and that’s when you get a vet out.”* - Competing amateur 1.

### Responses to colic

Once owners thought their horse had colic, three main strategies were identified: ‘wait and see’, initiate ‘lay treatment’ or ‘seek veterinary assistance’. None of these strategies were mutually exclusive and it was possible for an individual to adopt all three strategies over a period of time. The strategy adopted was dependent upon the interpretation of the severity of the colic, prior experience of colic and knowledge of the individual horse. One professional respondent emphasised the speed with which action needed to be taken with colic.

*”It can need treating very quickly if it’s severe... we would probably spot something at the uncomfortable stage before it gets to the severe stage but if we weren’t here all day, then you know things can have progressed a lot further on, by 12 or 8 hours later”* - Non-competing professional.

The transition between the different strategies occurred if colic was prolonged, or became progressively more severe. Lack of response to ‘lay treatments’ increased the chance of seeking veterinary assistance. Consequently, owners moved between the phases of, ‘wait and see’, ‘lay treatment’ and ‘seek veterinary assistance’ through monitoring and re-assessing the horse.

The ‘wait and see’ approach was used in different ways. On occasions, it was in response to an owner’s assessment that the colic was not very severe...

*”If it’s something just looking a bit uncomfortable we just monitor it.”* - Non-competing professional.

However, on other occasions it was reported as a routine occurrence and part of allowing time to decide whether veterinary assistance should be sought.

*”We’ll bring him (the horse) in we’ll leave him for an hour or so and then if he’s not any better we’ll get the vet.”* - Professional livery yard manager.

The length of time that owners would ‘wait and see’ varied dramatically from 10-15 minutes to a couple of hours with varying levels of monitoring in between.

‘Lay treatments’ were contingent upon the recognition of the observed signs as colic. Occasionally, lay treatments were used as a first line response if the owner considered the colic not to be severe enough to seek veterinary assistance straight away.

*”So I thought [...] I will give her a light bran mash, just to see because she is not showing signs of pain, she is just breathing heavily.”* - Amateur/hobbyist 3.

A variety of different ‘lay treatments’ were described by owners. By far the most common was ‘walking the horse’. It was believed the function of walking was to prevent the horse lying down and rolling. There was anxiety about rolling as it was believed that this may induce a twist in the gut leading to a fatal outcome.

*” I thought no he will stay on his feet and he will walk round this yard and you will not lie down and you will not roll [...] and I’ll keep him up and before the vet came he started to calm down.”* - Amateur pet/companion.

The action of walking the horse was also believed to calm the horse as well as being a source of distraction for the owner. However, although widely adopted, owners expressed considerable uncertainty about whether this was the right course of action for colic or not. Other forms of ‘lay treatment’ included altering the feed of the horse by removing or reducing feed, giving a soft bran mash or feed supplements.

The decision to call a veterinary surgeon involved many factors including; owners’ recognition and assessment of colic, assessment of responses to lay treatment, previous experience with colic, beliefs of what colic outcomes could be, the human-horse relationship and occasionally, the insurance status of the animal. Perceptions that the colic episode was severe and unlikely to be resolved (either spontaneously or through lay treatments) were likely to trigger seeking veterinary assistance. However, the veterinary surgeon was not always the preferred option; in one instance a respondent described that they would consult with one of their ‘mentors’ in preference to the veterinary surgeon.

“Not if you know what you are doing. Like (*name*) obviously does and I am certain if I had got one [horse] with it, I would call (*name*) before a vet.” - Male competing amateur.

Among other respondents, ‘mentors’ or knowledgeable horse-owners on livery yards were consulted during the assessment of the colic signs and influenced the decision to seek veterinary assistance.

### The veterinary-client relationship

Perceptions of the role of a veterinary surgeon influenced decisions of whether to seek veterinary assistance and what owners expected from such assistance.

*”It’s up to us as owners to make the decision what to do so it’s you know my say whether we call the vet or not and we have to tell the vets...whether we want it operating on or not and...whether we want the horse putting down.”* - Professional riding school manager.

While recognising the expertise of veterinary surgeons, some respondents were keen to emphasise the importance of the owners’ understanding and experience of the individual horse.

*” I think each person knows their own animal and probably better than the vet does, but you go to the vet and ask them for advice and then you formulate whether that advice is something.”* - Amateur/hobbyist 3.

Veterinary surgeons who demonstrated a caring nature, good inter-personal skills and willingness to monitor cases outside of normal working hours, were highly regarded. Being accessible by telephone was important to many owners. However, veterinary surgeons that appeared inconvenienced at being called out, dispassionate and hurried were not regarded so highly. Some owners appeared to have a vague understanding of the veterinary surgeon’s role in managing colic. Others appeared to have a strong comprehension of the typical process of the veterinary examination, assessment and treatment and even used medical terminology. The difference was often related to the frequency with which they had experienced colic and their knowledge of colic.

### Cost implications of veterinary treatment

Owners often made reference to the cost of veterinary intervention. This sometimes impacted upon the timing of the decision to seek veterinary assistance. Additionally, having an episode of colic on their records excluded future insurance claims. Secondary ‘costs’ of colic resulted from owners taking time off work to attend their horses. Colic surgery represented the most significant cost.

### Decisions surrounding surgery for colic

Colic surgery was a much-feared consequence of severe episodes of colic. Some, but not all owners, were aware that early veterinary intervention could improve surgical outcome. A number of contributory factors were involved in an owner’s decision to consent to surgery such as the age of the horse and fitness to travel.

*” Should it have been colic she [the vet] probably would have thought we’ll whizz him up to the surgery...but at 24 years old there was no way that was going to happen there was no way I could have transported him.”* - Amateur pet/companion.

Another significant factor was cost. Personal circumstances influenced the ability to raise funds for surgery. Sometimes the financial value or utility of the animal was evaluated against the cost of surgery (and post-surgical care) and could result in owners opting for euthanasia.

*“so he had to...have it operated on and he literally said how much is this gonna cost me? And they said well it could be 2 to 3 thousand and he’s like well I need to know which end of 2 to 3 thousand it’s going to be otherwise you know it’s me horses life here.”* - Professional livery yard manager

### Decision-making and euthanasia

In some colic cases, owners reported they had no choice but to opt for euthanasia, for example if the horse was not a candidate for surgery.

*”one of the vets came down and said there was nothing they could do, he wouldn’t eat, he was just lying down, he was getting up, he was rolling, throwing himself around and they basically just said he wouldn’t have survived the operation. He was in his 30s...so he was a little old to go through anything like that so they just put him to sleep.”* - Amateur breeder.

Alternatively, if the colic was considered too severe and not responsive to medication, the owner requested euthanasia as a kindness to the animal. Age and perceived quality of life were also involved in making this decision.

*”I remember feeling that I want this to end now, I don’t want him to sort of try to make it better...I knew, she was 30 anyway [...] there was no decision really she was going to die anyway.”* - Amateur pet/companion.

### Questionnaire results

In total, 722/1000 questionnaires were returned (49 were excluded due to: incorrect address; did not currently own horses; or were incomplete). 673 questionnaires contributed to the analysis (70.8% useable response rate). The majority of respondents (605, 89.9%) were female with only 45 (6.7%) male respondents (23 missing responses). The modal household income was £20,000-£29,999 *per annum*, with a right skewed distribution. The majority of respondents were in the higher earning brackets (see additional file 3).

In the main, respondents owned (or had primary responsibility for) one (219, 32.5%) or two horses (173, 25.7%). Four hundred (59.4%) respondents reported that their horses were insured for colic with 213 (31.6%) holding no insurance for colic. Of the 673 respondents, 419 stated they had owned a horse that had a history of colic (although this response is not representative of the general population due to the sampling frame). Among these respondents 50 (11.9% of 419 reporting colic experience) had horses that had experienced colic that had resolved spontaneously, 92 (22.0%) reported a single episode that resolved following veterinary prescribed medication, 29 (6.9%) had a horse with recurrent colic, 36 (8.6%) experienced a colic that required surgery and 38 (9.1%) experienced colic that resulted in the death of the horse. The remaining responses indicated a variety of colic episodes e.g.115 (27.4%) experienced both surgical colic and death of a horse.

### Owner typologies

Owner typologies were identified following cluster analysis of responses to 6 statements exploring the human-horse relationship.

• “I consider my horse/pony to be a pet” (Pet);

• “I consider my horses/ponies to be working animals” (Working animal);

• “Working with horses is part of my profession” (Profession),

• “I keep my horse for a sense of achievement (e.g. bringing on a youngster, becoming an accomplished rider etc)” (Achiever)

• “I keep horses for the satisfaction gained from the relationship I have with my horse” (Relator)

• “I keep horses in order to compete and win” (Sport)

**Figure 2:**
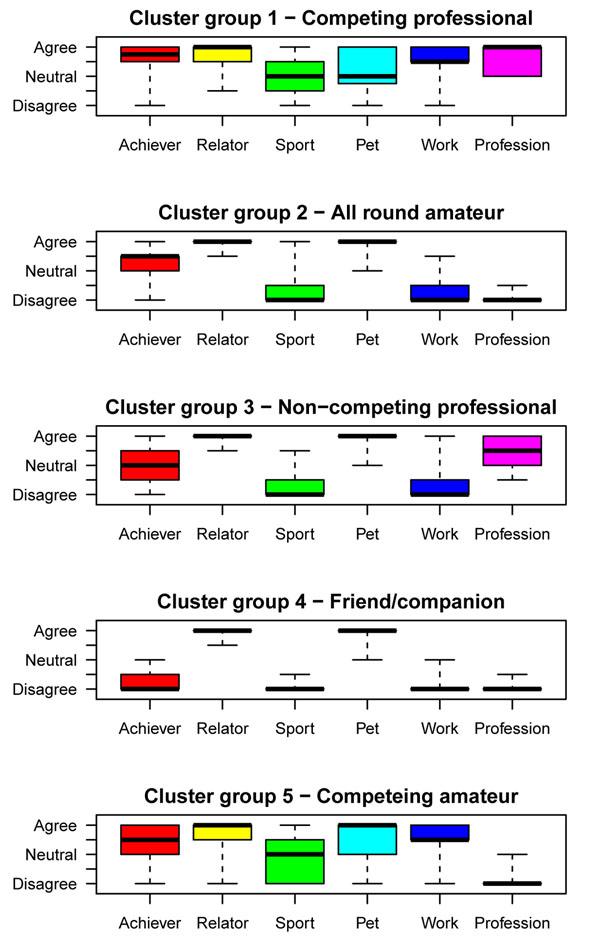
**Responses of each owner typology to the 6 questions used to create the typologies.** Each cluster group (cluster groups 1 to 5) is represented within a separate box-and-whisker plot. In each plot, the horizontal axes indicates each of the 6 questionnaire items used to measure aspects of the human-horse relationship (see supplementary information). The vertical axes indicate the strength of agreement with each item. The responses were recorded on a Likert-scale with categories Agree, Somewhat agree, Neutral, Somewhat disagree and Disagree (these were assigned a score of 1 for Agree through to 5 for Disagree). Some questionnaire items (‘achiever’, ‘relator’ and ‘sport’) were recorded on a 10-point scale (see supplementary information). These were converted to a 5-point scale and rounded up for the purposes of plotting. The box-and-whisker plots illustrate the distribution of responses among each cluster group with the boxes representing the median response (heavy black line) and first and third quartile (outer edges of the box), whiskers extend out to the minimum and maximum response. Hence, 25% of data lie between the box and each extreme. In instances where there was limited variation within the data, such that virtually all respondents gave the median response, only the median response is evident, and is shown by a heavy black line.

Cluster analysis revealed 5 meaningful owner typologies (Figure 2 and Table 1); 2 professional (clusters 1 and 3) and 3 amateur (Clusters 2, 4 and 5). Division into further sub-groups provided little additional insight. These 5 categories do not necessarily describe all respondents, but rather reflect some of the broad characteristics of the groups and may be useful conceptual tools to represent the diversity among horse-owners. The gender of the respondent was significantly associated with owner typology group (p=0.02; see additional file 3).

**Table 1:** Description of the 5 horse-owner typologies identified using cluster analysis of owners (n=623) responses to 6 questions investigating aspects of the horse-owner relationship.

Cluster number	Cluster name	Description
**1**	**Competing professional** **n=136**	**Professionals** predominantly saw their horses as working animals. This group reported a sense of achievement and satisfaction from their relationship with their horse, and competing and winning was often quite important to them. Many, but not all, felt their horse was also a pet.

**2**	**All round amateur** **n=209**	**Amateurs** their horses were reported as pets and they got a strong sense of satisfaction from their relationship with the horse, and a moderate sense of achievement. Sport tended not to be important and they strongly disagreed that their horses were working animals.

**3**	**Non-competing professional** **n=46**	**Professionals** differed from cluster 1 in that they strongly disagreed that competing and winning was important and disagreed that their horse was a working animal. They still felt their horses were pets and got a lot of satisfaction from their relationship with the horse, but had less sense of achievement from keeping horses.

**4**	**Friend/ Companion** **n=87**	**Amateurs** reported their horses were pets with which they strongly relate. Sport was not important, and their horse was not a working animal and they did not report a sense of achievement from owning the horse.

**5**	**Competing amateurs** **n=145**	**Amateurs** who competed and frequently saw their horses as working animals. Owning horses provided a sense of achievement and their relationship with the horse was moderately important.

### Role of the horse

The most frequently reported purpose for keeping horses was hacking/leisure followed by competition group 1 (Table 2). The role of the horse was significantly associated with owner typology group (see additional file 4).

**Table 2:** Horse usage categories and number (and %) of owners (n=623) responding to each category.

Role Category	Description	Number
Hack/Leisure		455 (73.0)

At Pasture		251 (40.3)

Breeding	Brood mares and stallions	78 (12.5)

Lessons	Gymkana, local shows, pony club activities, riding club activities, schooling	222 (35.6)

Competition 1	Dressage (below elementary level), driving (except in competitions), hunter trials/cross country, intro and unaffiliated eventing, jump cross, showing, show jumping	274 (44.0)

Competition 2	Dressage (elementary level and above), driving (in competitions), endurance rides (over 25 miles), hunting, pre-novice, novice and intermediate affiliated eventing	94 (15.1)

Competition 3	Advanced affiliated eventing	6 (1.0)

Competition 4	Racing, horse ball, hunter chasing, point to pointing, polo, polo crosse, team chasing	18 (2.9)

Other	Included rescue horses, in hand showing, natural horsemanship (Parelli methods) (n=6), police horse, RDA, side saddle / western riding, TREC and used at equine college.	21 (3.4)

### Colic signs and owner typology

**Figure 3:**
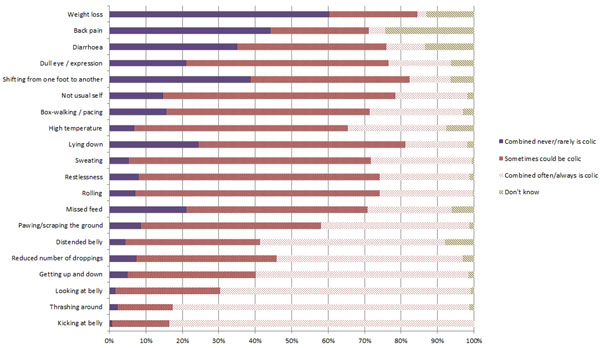
Responses for signs that indicated colic as perceived by owners.

Overall, signs that >50% of owners thought often or always indicated colic included: kicking at the belly; thrashing around; looking at the belly; getting up and down; reduced number of droppings; and, distended belly. Some signs were interpreted with less certainty with the majority of respondents indicating ‘sometimes could be colic’ (Figure 3). The only sign for which a significant difference was detected between owner typology groups was back pain (p=0.003; see additional file 5).

### Colic signs and decision to call the veterinary surgeon

**Figure 4:**
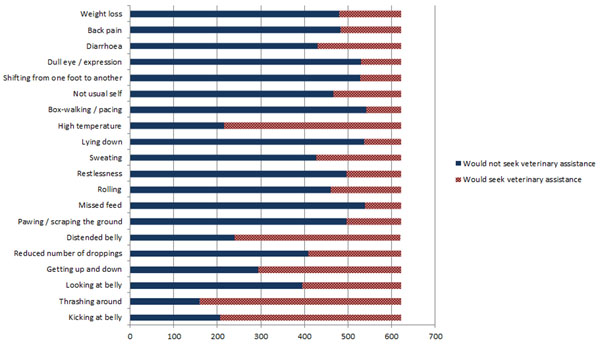
Behavioural signs and decision to call the veterinary surgeon.

Individual signs that prompted >50% of respondents to seek veterinary assistance included ‘getting up and down’, ‘distended belly’, ‘high temperature’, ‘kicking at belly’ and ‘thrashing around’ (Figure 4). The only clinical sign where there was a significant difference between owner groups was ‘high temperature’ (p = 0.004; see additional file 5).

### Assessment and management of colic episode versus owner typology

**Figure 5:**
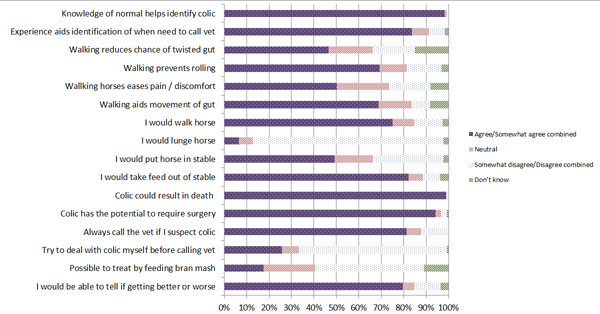
Responses to assessment and management of colic questions

The following elements were agreed by >50 % of the owners; knowledge of what’s normal helps to identify colic, experience aids identification of when veterinary assistance may be needed for colic, walking prevents a horse from rolling, walking eases pain and discomfort and aids movement of the gut, if a horse had colic they would walk the horse or put the horse in the stable and take feed out, colic could result in death of the horse or require surgery. Over 50% would always call a veterinary surgeon if they suspected colic and believed they could tell if the colic was getting better or worse (Figure 5).

There were significant differences between owner typology groups in the assessment and management of a colic episode including; knowledge that colic has the potential to require surgery (p=0.05), beliefs about walking horses and its purpose to prevent rolling (p=0.04), and to reduce the chance of a twisted gut (p=0.03), and owner’s experience of identifying when veterinary assistance is required (p<0.001; see additional file 6).

### Decisions surrounding veterinary treatment and owner typology

**Figure 6:**
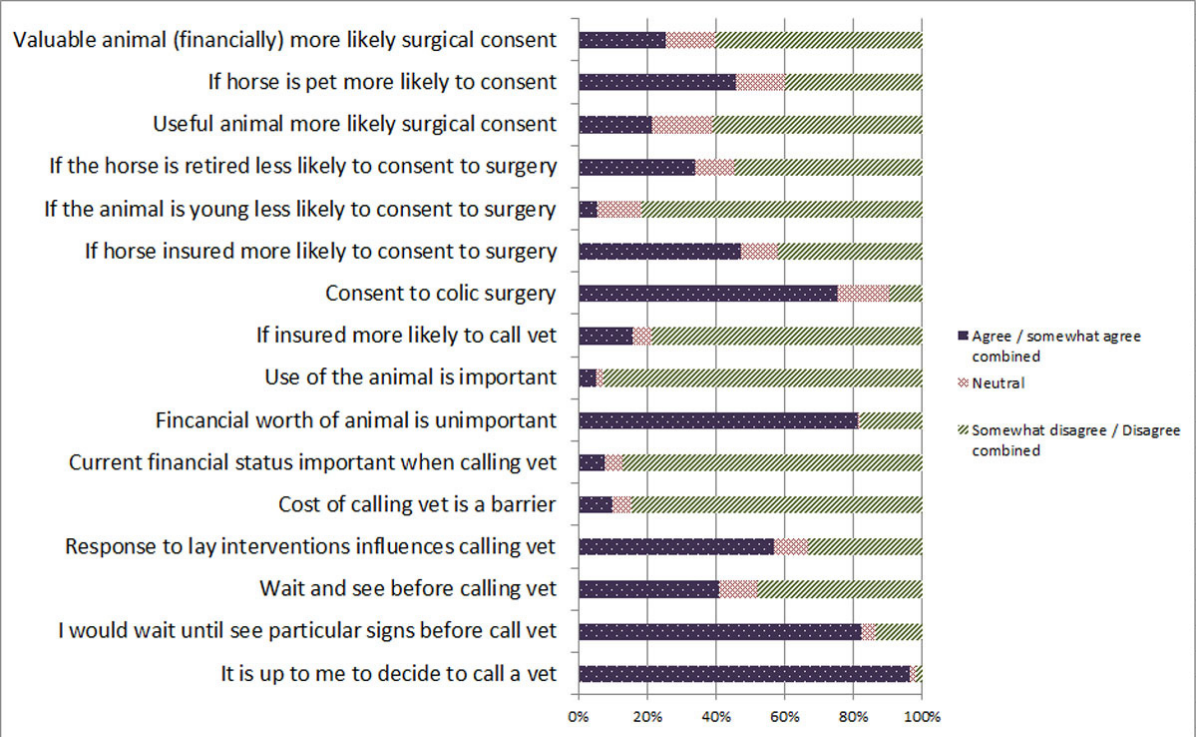
Responses to decisions surrounding veterinary treatment

In general, >50% of respondents disagreed with the following statements: they would be more likely to consent to surgery if the horse was financially valuable or if the horse was well adapted for its use; they would be unlikely to consent to surgery if the horse was retired or young; their horses insurance status influenced seeking veterinary assistance; the use of the animal had an important influence on the decision to seek veterinary assistance; and, the cost of calling the veterinary surgeon was a barrier to seeking assistance (Figure 6).

Over 50% of respondents agreed they would consent to colic surgery if their veterinary surgeon recommended it. How the horse responded to their actions had an influence on whether to call the veterinary surgeon, whereas financial worth was an unimportant factor. Most agreed they would wait until they saw particular signs before calling the veterinary surgeon and that it was up to them to make the decision (Figure 6). There were significant differences between the owner typology groups and their decisions about veterinary treatment. These included; how the horse responded to their actions (p=0.007), their current financial status (p=0.03), if the horse was insured (p=0.006) and decisions surrounding colic surgery including if the horse was insured (p=0.02), retired (p<0.001), well adapted for its use (p=0.001) or financially valuable (p<0.001; see additional file 7).

## Discussion

The mixed-method approach allowed a detailed exploration of horse-owners’ understanding and management of colic. The findings illustrate the value of undertaking qualitative research and demonstrate the complex components of decision-making. The questionnaire study examined these factors within a larger population.

Davison *et al. *[17] acknowledged that people outside of the medical community construct meanings of illness through lay interpretations of symptomatology, aetiology and epidemiology. This study identified signs (both objective and subjective) that horse-owners’ associated with colic, and described lay beliefs regarding the management and treatment of a horse with colic. It is recognised within medical sociology that lay beliefs have an important impact upon the way patients frame their illness and manage advice and treatment [18-21]. This study suggests that horse-owners’ views and knowledge of colic have a direct impact on when veterinary intervention is sought.

Generally, responses indicated good knowledge of signs that could be attributed to colic and these did not differ significantly between owner typology groups. However, there were differences in the certainty with which respondents attributed these signs to colic. This may be because some of the listed behaviours could, in a different context, be viewed as normal (e.g. rolling, pawing, sweating). Triggers for seeking veterinary assistance were explored both qualitatively and within the questionnaire. Lay interpretation of colic severity and subsequent timing of seeking veterinary assistance could have important implications for colic outcomes.

This study reports a range of objective and subjective assessments that owners use in determining their horse’s health, and deviations from these alerted owners to a change in health status. Buckley *et al.*[7] reported a variety of signs that owners interpreted as a healthy horse (e.g. bright eyes, shiny coat, good body condition). However, the accuracy with which owners are able to detect changes in their horse’s health has been debated. Within a geriatric horse population [22], owner-reported signs were generally not in agreement with veterinary findings for a wide number of disease presentations. It was hypothesised that owners were more likely to attribute observed changes to normal ageing and may be less sensitive to changes in horses retired from work. However, in some diseases, strong correlations between owner-reported and clinical findings have been demonstrated reinforcing the importance of owners in the diagnosis and treatment of their horses [23].

In a study of heart disease in people, a constellation of symptoms were hypothesised to contribute to the decision to seek medical care [24]. These were prefaced by initial symptoms that were mild and did not trigger help-seeking [24]. The ‘wait and see’ and ‘lay treatment’ phases of the colic management model were also evident in this study of human behaviour [24]. The authors suggested that self-monitoring and treating of symptoms, consulting friends, relatives or other medical personnel were all influential in increasing or delaying the time taken to consult a professional [24]. Within the colic management model, the ‘wait and see’ strategy was moderated by interpretation and reassessment of signs, (sometimes occurring in consultation with friends or equine ‘mentors’), and the horse’s response to ‘lay treatments’. In the context of heart attack, the authors [24] argue that seeking medical attention on the basis of early recognition of signs improves outcomes and prognosis, similar to the benefits associated with the early recognition and treatment of colic [6].

Owner knowledge and experience with colic developed within a social context (such as that found within a livery yard [25]), and was assimilated from many sources. Other studies have reported that lay sources of information were often the first point of reference for their ponies’ health, rather than the veterinary surgeon [7] and [26]. However, a recent study demonstrated differences between owner groups and the information sources they accessed in different situations [27], highlighting the complexity surrounding information seeking and the assimilation of knowledge.

Conrad & Barker [28], in their examination of how people understand illness, identified three main premises; some illnesses are embedded in cultural meaning; all illnesses are socially constructed at the experiential level (i.e. how an individual understands and lives with illness), and; medical knowledge is not ‘given’ by nature but is constructed and developed by individuals. Based upon the current findings, we theorise that the meaning assigned to colic varies in all these three domains. Owners’ interpretation and decision making in response to colic drew from lay knowledge, personal experience and the experience of others. These findings have implications for how colic management advice is received, understood and acted upon by owners.

Veterinary-client communication was particularly important in supporting owners through decision-making during a colic episode. The approach a veterinary surgeon adopts may vary between clients according to the prior knowledge, experience and attitudes of the horse-owner. As Weiner [29] highlights, a veterinary surgeon’s role shifts in line with the contextual and situational needs of clients. In this study, owners’ views about veterinary assistance were influenced not just by the medical management of the horse but by the veterinary surgeon’s manner, their approach to assisting owners with difficult decisions (e.g. consenting to surgery or euthanasia) and the owners’ experience of the visit.

The role of finance upon seeking veterinary help was varied. The respondents belonged to households with above average (for their location) household incomes (£20,000-£29,999 *per annum* as opposed to ≤ £14,000 *per annum*, Office for National Statistics 2007 [30]). While many horse-owners fund their equestrian pursuits at the expense of holidays, entertainment or clothes [31], unexpected emergencies such as colic, may stretch available budgets. In the qualitative study, the cost of veterinary assistance and treatment were reported to influence the timing of the decision to call the veterinary surgeon and consenting to surgery. However, in the questionnaire, money was not a significant factor in seeking veterinary assistance. Lane and Whigham [32] also reported few owners citing cost as a deciding factor in the treatment of colic. There are a number of possible explanations for this apparent discrepancy. Making decisions about ‘health’ based on finance may be viewed as antithetical to the management of a much-loved pet, and the different research techniques may permit this to be explored to different levels. Qualitative data allows a fuller explanation and enables respondents to contextualise the role of finance in their decisions. Whereas pre-defined questionnaire categories may restrict respondents’ ability to appropriately represent their views. Facing this constraint within a questionnaire, respondents may be more inclined to reject the role of finance in their decisions. Further examination of the dynamic socio-economic context of equine management and its impact on equine health is an area worthy of further investigation.

The possibility for selection bias within this study is acknowledged as the sampling frame largely comprised respondents who had either previously been involved with research or had accessed veterinary services for colic treatment. It may be that owner knowledge and decision making reflected within this population is not comparable to other populations, particularly those with less frequent contact with the veterinary profession. Furthermore, among owners who consented to an in-depth interview, nearly half had experienced death of a horse due to colic. This may have contributed to their motivation to take part and represents a potential responder bias. However, it is considered that a balanced and diverse array of experiences and opinions were represented. Additionally, among the questionnaire respondents only 9% had lost a horse due to colic and many respondents did not have previous experience of colic. The affiliations of the researchers were known to respondents and therefore may have influenced responses relating to views on accessing veterinary services. However, this did not seem to deter participants from providing open responses within the interview. The positionality of the primary researcher as a veterinary surgeon and researcher may, to some extent, have influenced the interpretation of the data, although this insight was likely to further support understanding of owner narratives of colic.

The equine industry comprises a diverse spectrum of equestrian activities [4,33]. Although our sample included a cross-section of equestrian disciplines, it was predominantly drawn from the leisure rider population (comparable with other regional studies [12,34]). Our owner typology groups attempted to classify owners by a number of factors, and included 2 ‘professional’ and 3 groups of ‘amateur’ horse-owners. Subsequent analyses illustrated where these groups responded differently and could be reflective of differing owner knowledge, confidence and motivations for decision-making regarding colic. We do not suggest that this typology provides definitive and mutually exclusive owner types, and, as Jones [16] also notes, horse-owners may belong to more than one group. However, these categories may serve to conceptualise groups of horse-owners and contribute to tailoring information for veterinary clients.

## Conclusions

This study provides an in-depth description of how horse-owners from the North-West of the UK understand and manage colic and provides important new insights into the actions and decisions made prior to calling the veterinary surgeon. The mixed method approach allowed a broad illustration of how colic is viewed within a diverse population of horse-owners. The findings may serve as a basis upon which to tailor existing programmes designed to educate owners about colic management strategies.

## Competing interests

The authors declare that they have no competing interests.

## Authors’ contributions

All authors were involved in the conception and design of the study. CS conducted the interviews, questionnaire design, and analysis and had a primary role in preparing the manuscript. EP assisted in the qualitative analysis. RC and EP supervised the project and contributed to questionnaire design, analysis and drafting of the manuscript. All authors contributed to reviewing the final manuscript. All authors read and approved the final manuscript.

## Endnotes

^1^ Throughout the manuscript the term ‘medical colic’ refers to a colic episode that resolves with conservative management, with or without medication, and ‘surgical colic’ refers to a colic episode from which the horse would not survive without surgical intervention.

^2^ Where [...] is presented within the written quotes, this indicates where words have been omitted (in order to reduce repetition or colloquialisms). In all cases the original meaning has been maintained.

## Supplementary Material

Scantlebury additional file 1 Questionnaire items exploring the human-horse relationship. Click here for file

Scantlebury additional file 2 Further details of participants from the in-depth interviews (15 participants). Click here for file

Scantlebury additional file 3 Further demographic information about the respondents. Click here for file

Scantlebury additional file 4 Table reporting associations between the role of the horse and the owner typology. Click here for file

Scantlebury additional file 5 Tables reporting behavioural signs and recognition of colic versus owner typology group and reporting associations between behavioural signs and the decision to seek veterinary assistance. Click here for file

Scantlebury additional file 6 Assessment and management of colic episode versus owner typology group. Click here for file

Scantlebury additional file 7 Decisions surrounding veterinary treatment and owner typology group. Click here for file

## Declarations

We acknowledge the BEVA colic symposium for funding the publishing costs of this article.

This article has been published as part of *BMC Veterinary Research* Volume 10 Supplement 1, 2014: Selected articles from the Eleventh International Equine Colic Research Symposium. The full contents of the supplement are available online at http://www.biomedcentral.com/bmcvetres/supplements/10/S1. Publication of this supplement has been funded by The British Equine Veterinary Association.
