# Supplementary material for: Could it be colic? Horse-owner decision making and practices in response to equine colic
Source: BMC Vet Res. 2014 Jul 7;10(Suppl 1):S1. doi: 10.1186/1746-6148-10-S1-S1 (PMC4122872; doi:10.1186/1746-6148-10-S1-S1)
Supplement: Scantlebury additional file 1 — Questionnaire items exploring the human-horse relationship. [file 1746-6148-10-S1-S1-S1.PDF]

Additional file 1:

Questionnaire items exploring the human-horse relationship

Questions on the human-horse relationship included 3 descriptors inspired by Jones, B. (1983), ‘Achiever’, ‘Relator’ and ‘Riding is a Sport’. An image from the questionnaire is included below which details how these categories were defined and recorded. NB: The item ‘hobby/passion’ was answered similarly by most respondents and did not have any discriminatory power within the cluster analysis and therefore was excluded.

Section 6: Human - Horse Relationship

14. Please indicate along the scale how you view your horses.

	Agree	Somewhat agree	Neutral	Somewhat disagree	Disagree
I consider my horse / pony (s) to be pets	☐	☐	☐	☐	☐
I consider my horse / pony (s) to be working animals (e.g. for competition, financial gain etc.)	☐	☐	☐	☐	☐
Working with horses is part of my profession	☐	☐	☐	☐	☐
Horses are a hobby / passion of mine	☐	☐	☐	☐	☐

15. For each of the categories below, please indicate along the scale where you place yourself as a horseman / horsewoman and your relationship with horses.

1 = disagree strongly **10 = strongly agree**

		1	2	3	4	5	6	7	8	9	10
**Achiever**	I keep horses for the sense of achievment (e.g. bringing on a youngster, becoming an accomplished rider etc.)	☐	☐	☐	☐	☐	☐	☐	☐	☐	☐
**Relator**	I keep horses for the satisfaction gained from the personal relationship I have with my horse	☐	☐	☐	☐	☐	☐	☐	☐	☐	☐
**Riding is a sport**	I keep horses in order to compete and win	☐	☐	☐	☐	☐	☐	☐	☐	☐	☐

31177
