# Supplementary material for: Could it be colic? Horse-owner decision making and practices in response to equine colic
Source: BMC Vet Res. 2014 Jul 7;10(Suppl 1):S1. doi: 10.1186/1746-6148-10-S1-S1 (PMC4122872; doi:10.1186/1746-6148-10-S1-S1)
Supplement: Scantlebury additional file 2 — Further details of participants from the in-depth interviews (15 participants). [file 1746-6148-10-S1-S1-S2.PDF]

Additional file 2

Further details of participants from the in-depth interviews (15 participants).

Professional owners included one owner/manager of a breaking in yard, one riding school manager and riding instructor, one livery yard manager, and one owner of a small-scale livery yard involved with point-to-pointing, eventing and breeding. The amateur group included three individuals who competed in local level competitions, four who used their horses purely for hacking, one owner who kept horses due to their daughter's interest, one amateur breeder who did not ride their horses, one owner who had retired hunters and one who took riding lessons.) Participants also varied by their experience of colic. Four owners had no direct personal experience of colic. Three owners had owned horses that had experienced a single episode of medical colic. Seven owners had experienced owning a horse with recurrent colic, 1 with recurrent medical colic, 4 underwent surgery for colic (3 subsequently died, and one continued to have recurrent colic) and two that experienced recurrent medical colic and subsequent death due to colic. One owner had experienced a single episode of colic that required surgery but the horse subsequently died due to colic.
