# Supplementary material for: Could it be colic? Horse-owner decision making and practices in response to equine colic
Source: BMC Vet Res. 2014 Jul 7;10(Suppl 1):S1. doi: 10.1186/1746-6148-10-S1-S1 (PMC4122872; doi:10.1186/1746-6148-10-S1-S1)

Additional file 3:

Further demographic information on the questionnaire respondents

The modal age category of females was slightly lower than male respondents. (41-50 years females and 51-60 years males).

Age distribution of respondents split by gender. Note different scales on x-axes.

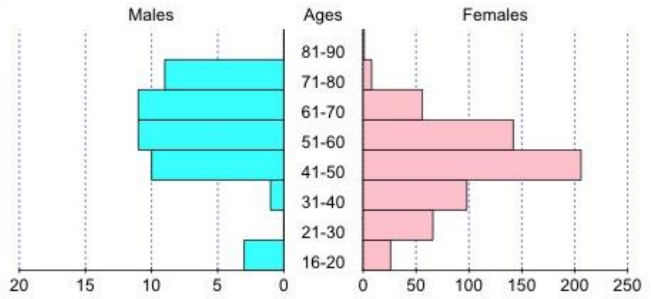

Approximately one-third of respondents were in full-time permanent employment (247, 36.7%) and this was significantly associated with where they kept their horses in relation to their home premises (p= 0.03) and the type of livery paid for (p = 0.03) (supplementary information).

Relationship between full time / part time employment status and where horses are kept in relation to home premises

	Total (n)	Kept at home (%)	Away from home and up to 1 mile away (%)	>1mile up to 5 (%)	>5miles up to 10 (%)	>10miles up to 15 (%)	>15miles away (%)
Part time employment	169	37.3	21.3	29.0	10.1	1.2	1.2
Full time employment	389	32.9	13.1	35.0	12.3	4.9	1.8

Chi-square p=0.03

Relationship between full time / part time employment status and livery basis

	Total (n)	Not applicable (%)	Full livery (%)	Part livery (%)	DIY livery (%)	Other (%)
Part time	145	3.4	3.4	42.8	42.8	7.6
Full time	345	7.5	8.1	45.5	31.3	7.5

Chi-square p=0.03

Association between respondent gender and typology group

	All	Competing professional (%)	All round amateur (%)	Non competing Professional (%)	Friend / companion (%)	Competing amateurs (%)	Chi-square P
Female	563	86.8	90.0	95.7	95.4	89.7	
Male	40	11.8	3.8	4.3	2.3	8.3	0.02

Total household income, frequencies.

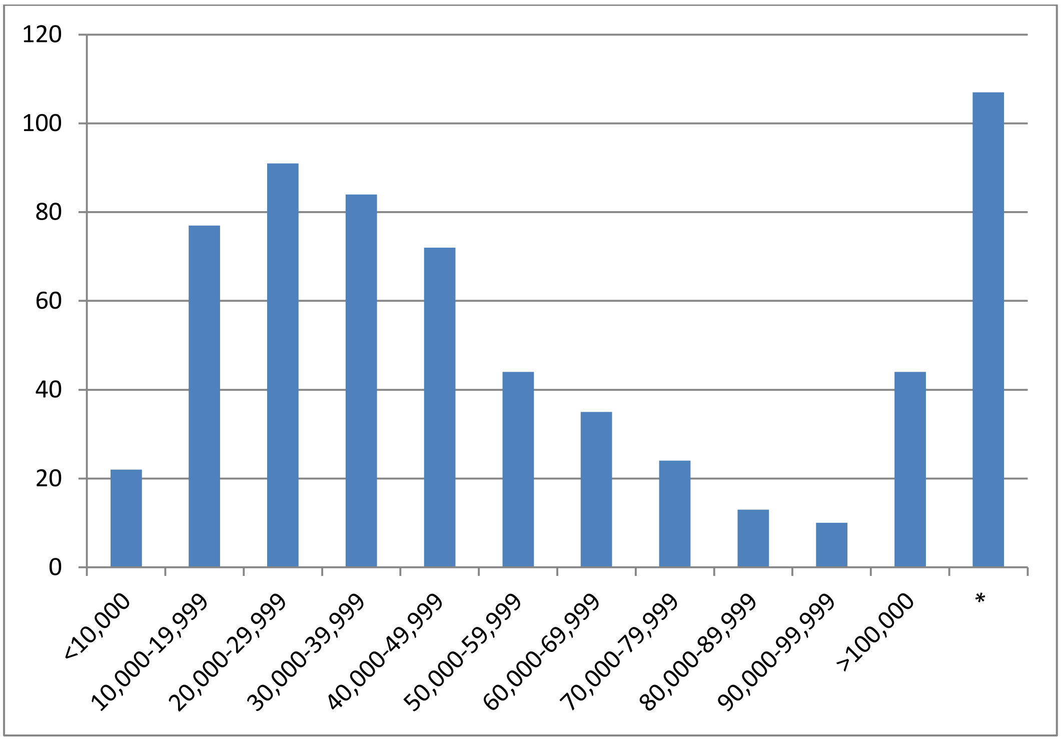

Supplement: Scantlebury additional file 3 — Further demographic information about the respondents. [file 1746-6148-10-S1-S1-S3.PDF]
