# Supplementary material for: Could it be colic? Horse-owner decision making and practices in response to equine colic
Source: BMC Vet Res. 2014 Jul 7;10(Suppl 1):S1. doi: 10.1186/1746-6148-10-S1-S1 (PMC4122872; doi:10.1186/1746-6148-10-S1-S1)
Supplement: Scantlebury additional file 4 — Table reporting associations between the role of the horse and the owner typology. [file 1746-6148-10-S1-S1-S4.PDF]

Additional file 4:

Associations between the role of horse and the owner typology

Role of horse		Total	Competing professional (%)	All round amateur (%)	Non competing Professional (%)	Friend / companion (%)	Competing amateurs (%)	Chi-square p
Lessons	No	412	61.2	65.6	65.2	86.2	58.6	
	Yes	214	38.8	34.4	34.8	13.8	41.4	<0.001
At pasture	No	377	50.4	64.6	52.2	54.0	69.7	
	Yes	249	49.6	35.4	47.8	46.0	30.3	0.004
Competition 1	No	363	41.0	65.1	67.4	83.9	45.5	
	Yes	263	59.0	34.9	32.6	16.1	54.5	<0.001
Competition 2	No	532	70.5	94.7	91.3	98.9	74.5	
	Yes	94	29.5	5.3	8.7	1.1	25.5	<0.001
Competition 3	No	620	96.4	100.0	100.0	100.0	99.3	
	Yes	6	3.6	0.0	0.0	0.0	0.7	*
Competition 4	No	608	92.1	99.0	100.0	100.0	96.6	
	Yes	18	7.9	1.0	0.0	0.0	3.4	<0.001
Hack	No	176	43.9	18.2	15.2	18.4	37.2	
	Yes	450	56.1	81.8	84.8	81.6	62.8	<0.001
Breeding	No	547	68.3	96.2	87.0	95.4	88.3	
	Yes	79	31.7	3.8	13.0	4.6	11.7	<0.001
