# Supplementary material for: Could it be colic? Horse-owner decision making and practices in response to equine colic
Source: BMC Vet Res. 2014 Jul 7;10(Suppl 1):S1. doi: 10.1186/1746-6148-10-S1-S1 (PMC4122872; doi:10.1186/1746-6148-10-S1-S1)
Supplement: Scantlebury additional file 5 — Tables reporting behavioural signs and recognition of colic versus owner typology group and reporting associations between behavioural signs and the decision to seek veterinary assistance. [file 1746-6148-10-S1-S1-S5.PDF]

Additional file 5: Behavioural signs and recognition of colic versus owner typology

	Typology group †	Total	N / R	S colic	O / A	Don't know	Don't know (n)	Chi square	p
Rolling	1	132	6.8	66.7	26.5	*			
	2	202	4.0	70.8	25.2	*			
	3	45	6.7	57.8	35.6	*			
	4	85	8.2	62.4	29.4	*			
	5	141	11.3	68.8	19.9	*	3	11.947	0.2
Sweating	1	130	6.9	67.7	25.4	*			
	2	200	2.5	71.0	26.5	*			
	3	44	2.3	63.6	34.1	*			
	4	81	6.2	56.8	37.0	*			
	5	142	8.5	66.2	25.4	*	4	13.034	0.1
Looking at belly	1	131	1.5	26.7	71.8	*			
	2	201	1.0	33.3	65.7	*			
	3	45	4.4	26.7	68.9	*			
	4	83	2.4	22.9	74.7	*			
	5	140	1.4	28.6	70.0	*	6	*	*
Kicking at belly	1	132	0.8	15.2	84.1	*			
	2	202	0.5	14.4	85.1	*			
	3	45	4.4	11.1	84.4	*			
	4	85	0.0	14.1	85.9	*			
	5	142	0.0	21.1	78.9	*	1	*	*
Pawing / scraping ground	1	132	6.8	48.5	44.7	*			
	2	197	10.2	51.3	38.6	*			
	3	43	9.3	41.9	48.8	*			
	4	82	11.0	42.7	46.3	*			
	5	140	7.1	56.4	36.4	*	8	7.28	0.5
Thrashing around	1	131	2.3	13.7	84.0	*			
	2	198	0.5	16.2	83.3	*			
	3	43	7.0	4.7	88.4	*			
	4	81	4.9	12.3	82.7	*			
	5	141	1.4	20.6	78.0	*	8	*	*
Box walking / pacing	1	129	17.1	59.7	23.3	*			
	2	195	13.8	63.1	23.1	*			
	3	42	23.8	42.9	33.3	*			
	4	79	21.5	48.1	30.4	*			
	5	137	13.1	56.9	29.9	*	18	11.435	0.2
Lying down	1	128	31.3	53.9	14.8	*			
	2	197	24.9	57.4	17.8	*			
	3	45	15.6	55.6	28.9	*			

	4	84	23.8	59.5	16.7	*			
	5	139	23.0	61.9	15.1	*	12	8.619	0.4
Restlessness	1	131	9.2	64.9	26.0	*			
	2	196	8.2	68.9	23.0	*			
	3	45	6.7	64.4	28.9	*			
	4	84	10.7	64.3	25.0	*			
	5	136	5.9	67.6	26.5	*	7	2.934	1
Getting up and down	1	133	4.5	33.1	62.4	*			
	2	197	4.6	36.0	59.4	*			
	3	43	7.0	30.2	62.8	*			
	4	82	6.1	37.8	56.1	*			
	5	140	4.3	38.6	57.1	*	9	2.372	1
Missed feed	1	131	24.4	49.6	22.9	3.1	*		
	2	197	19.8	51.8	23.9	4.6	*		
	3	45	28.9	42.2	17.8	11.1	*		
	4	84	19.0	42.9	28.6	9.5	*		
	5	141	18.4	53.2	20.6	7.8	*	13.327	0.4
Reduced number of droppings	1	131	9.2	39.7	51.1	*			
	2	194	7.2	34.5	58.2	*			
	3	43	9.3	37.2	53.5	*			
	4	81	6.2	42.0	51.9	*			
	5	138	6.5	46.4	47.1	*	19	6.058	0.6
Not usual self	1	128	13.3	62.5	24.2	*			
	2	201	17.9	61.7	20.4	*			
	3	44	20.5	63.6	15.9	*			
	4	81	9.9	70.4	19.8	*			
	5	140	13.6	68.6	17.9	*	11	6.824	0.6
Dull eye / expression	1	130	23.1	56.2	18.5	2.3	*		
	2	202	20.8	53.5	18.3	7.4	*		
	3	44	25.0	56.8	9.1	9.1	*		
	4	83	18.1	57.8	13.3	10.8	*		
	5	141	19.9	56.0	19.1	5.0	*	12.577	0.4
Shifting from one foot to another	1	131	44.3	42.0	9.9	3.8	*		
	2	201	36.8	44.3	11.9	7.0	*		
	3	45	51.1	33.3	2.2	13.3	*		
	4	84	38.1	44.0	11.9	6.0	*		
	5	142	32.4	47.2	14.1	6.3	*	16.502	0.2
Weight loss	1	130	69.2	22.3	1.5	6.9	*		
	2	200	58.5	24.5	2.5	14.5	*		
	3	45	62.2	24.4	2.2	11.1	*		
	4	83	59.0	22.9	2.4	15.7	*		
	5	142	54.2	26.1	4.2	15.5	*	11.053	0.5
High temperature	1	134	11.2	57.5	24.6	6.7	*		
	2	203	8.4	56.7	25.1	9.9	*		

	3	45	6.7	68.9	22.2	2.2	*		
	4	87	2.3	56.3	32.2	9.2	*		
	5	142	3.5	59.9	30.3	6.3	*	17.8	0.1
Diarrhoea	1	130	41.5	44.6	6.9	6.9	*		
	2	204	36.8	39.7	8.3	15.2	*		
	3	45	33.3	40.0	11.1	15.6	*		
	4	84	29.8	39.3	11.9	19.0	*		
	5	140	30.7	39.3	16.4	13.6	*	18.069	0.1
Back pain	1	131	53.4	29.8	3.8	13.0	*		
	2	201	42.8	22.9	5.5	28.9	*		
	3	44	25.0	27.3	4.5	43.2	*		
	4	83	38.6	31.3	1.2	28.9	*		
	5	134	47.0	27.6	5.2	20.1	*	30.318	0.003
Distended belly	1	134	9.0	31.3	54.5	5.2	*		
	2	204	3.4	39.7	49.5	7.4	*		
	3	45	4.4	37.8	48.9	8.9	*		
	4	85	3.5	35.3	51.8	9.4	*		
	5	139	2.2	38.1	48.9	10.8	*	12.762	0.4

*Algorithm would not converge

†Typology groups; 1= Competing professional, 2= All round amateur, 3= Non-competing professional, 4= Friend/companion, 5= Competing amateurs

N/R= combined categories for 'never' or 'rarely' , S Colic='sometimes' indicates colic, O/R= combined categories 'often' and 'always' indicates colic.

Behavioural signs and decision to call the veterinary surgeon versus owner typology group.

			**Indicated would call vet**			
**Sign**	**Typology†**	**Total**	**No (%)**	**Yes (%)**	**Chi square**	**p**
Rolling	1	136	77.2	22.8		
	2	208	71.2	28.8		
	3	46	69.6	30.4		
	4	87	70.1	29.9		
	5	144	79.2	20.8	4.814	0.3
Sweating	1	136	75.0	25.0		
	2	208	68.3	31.7		
	3	46	65.2	34.8		
	4	87	60.9	39.1		
	5	144	68.8	31.3	5.242	0.3
Looking at belly	1	136	59.6	40.4		
	2	208	63.5	36.5		
	3	46	58.7	41.3		
	4	87	59.8	40.2		

	5	144	71.5	28.5	6.018	0.2
Kicking at belly	1	136	39.7	60.3		
	2	208	27.4	72.6		
	3	46	32.6	67.4		
	4	87	32.2	67.8		
	5	144	36.8	63.2	6.651	0.2
Pawing	1	136	73.5	26.5		
	2	208	81.7	18.3		
	3	46	76.1	23.9		
	4	87	78.2	21.8		
	5	144	85.4	14.6	7.157	0.2
Thrashing around	1	136	33.1	66.9		
	2	208	23.1	76.9		
	3	46	15.2	84.8		
	4	87	28.7	71.3		
	5	144	23.6	76.4	8.147	0.09
Box-walking / pacing	1	136	87.5	12.5		
	2	208	89.4	10.6		
	3	46	82.6	17.4		
	4	87	85.1	14.9		
	5	144	86.1	13.9	2.263	0.7
Lying down	1	136	86.8	13.2		
	2	208	85.1	14.9		
	3	46	80.4	19.6		
	4	87	85.1	14.9		
	5	144	91.7	8.3	5.528	0.2
Restlessness	1	136	80.9	19.1		
	2	208	78.4	21.6		
	3	46	80.4	19.6		
	4	87	80.5	19.5		
	5	144	80.6	19.4	0.446	1
Getting up and down	1	136	46.3	53.7		
	2	208	49.0	51.0		
	3	46	47.8	52.2		
	4	87	46.0	54.0		
	5	144	46.5	53.5	0.404	1
Missed feed	1	136	90.4	9.6		
	2	208	85.1	14.9		
	3	46	87.0	13.0		
	4	87	81.6	18.4		
	5	144	88.9	11.1	4.658	0.3
Reduced number of droppings	1	136	64.7	35.3		

	2	208	64.4	35.6		
	3	46	67.4	32.6		
	4	87	67.8	32.2		
	5	144	67.4	32.6	0.613	1
Not usual self	1	136	77.2	22.8		
	2	208	72.6	27.4		
	3	46	78.3	21.7		
	4	87	74.7	25.3		
	5	144	75.7	24.3	1.298	0.9
Dull eye / expression	1	136	86.8	13.2		
	2	208	83.2	16.8		
	3	46	93.5	6.5		
	4	87	85.1	14.9		
	5	144	84.7	15.3	4	0.4
Shifting from one foot to another	1	136	87.5	12.5		
	2	208	83.7	16.3		
	3	46	84.8	15.2		
	4	87	81.6	18.4		
	5	144	87.5	12.5	2.456	0.7
Weight loss	1	136	80.1	19.9		
	2	208	77.9	22.1		
	3	46	73.9	26.1		
	4	87	77.0	23.0		
	5	144	75.0	25.0	1.408	0.8
High temperature	1	136	43.4	56.6		
	2	208	34.1	65.9		
	3	46	15.2	84.8		
	4	87	39.1	60.9		
	5	144	29.9	70.1	15.467	0.004
Diarrhoea	1	136	72.8	27.2		
	2	208	69.2	30.8		
	3	46	56.5	43.5		
	4	87	64.4	35.6		
	5	144	72.9	27.1	5.994	0.2
Back pain	1	136	83.8	16.2		
	2	208	76.4	23.6		
	3	46	71.7	28.3		
	4	87	73.6	26.4		
	5	144	78.5	21.5	5.122	0.3
Distended belly	1	136	42.6	57.4		

	2	208	38.5	61.5		
	3	46	21.7	78.3		
	4	87	41.4	58.6		
	5	144	38.9	61.1	7.203	0.1

†Typology groups; 1= Competing professional, 2= All round amateur, 3= Non-competing professional, 4= Friend/companion, 5= Competing amateurs
