# Supplementary material for: Could it be colic? Horse-owner decision making and practices in response to equine colic
Source: BMC Vet Res. 2014 Jul 7;10(Suppl 1):S1. doi: 10.1186/1746-6148-10-S1-S1 (PMC4122872; doi:10.1186/1746-6148-10-S1-S1)
Supplement: Scantlebury additional file 6 — Assessment and management of colic episode versus owner typology group. [file 1746-6148-10-S1-S1-S6.PDF]

Additional file 6: Assessment and management of colic episode versus owner typology

	Typology†	Total	A/SA (%)	N/Don't know (%)	SD/D (%)	Chi square	p
I would be able to tell if it was getting better or worse	1	134	84.3	6.7	9.0		
	2	205	75.6	9.3	15.1		
	3	46	93.5	2.2	4.3		
	4	86	73.3	11.6	15.1		
	5	144	80.6	9.0	10.4	13.607	0.09
Treat by feeding bran mash	1	134	17.9	35.8	46.3		
	2	206	15.5	30.6	53.9		
	3	46	26.1	32.6	41.3		
	4	85	16.5	32.9	50.6		
	5	143	18.2	37.8	44.1	6.263	0.6
I would try to deal with colic myself before calling a vet	1	133	30.1	10.5	59.4		
	2	204	22.5	6.9	70.6		
	3	45	26.7	11.1	62.2		
	4	87	20.7	5.7	73.6		
	5	143	29.4	7.7	62.9	8.402	0.4
I would always call the vet if I suspect colic	1	134	78.4	8.2	13.4		
	2	205	83.4	5.9	10.7		
	3	45	75.6	13.3	11.1		
	4	87	89.7	3.4	6.9		
	5	143	78.3	4.9	16.8	11.76	0.2
Colic has the potential to require surgery	1	136	92.6	2.2	5.1		
	2	206	94.7	2.4	2.9		
	3	46	97.8	0.0	2.2		
	4	85	96.5	2.4	1.2		
	5	144	92.4	6.9	0.7	15.378	0.05
Colic could result in the death of the horse	1	135	97.0	1.5	1.5		
	2	206	99.0	0.0	1.0		
	3	46	100.0	0.0	0.0		
	4	87	98.9	0.0	1.1		
	5	144	100.0	0.0	0.0	*	*
**If my horse had colic...**							
...I would take all feed out of the stable	1	136	89.0	6.6	4.4		
	2	204	78.4	13.2	8.3		
	3	46	82.6	10.9	6.5		
	4	86	80.2	14.0	5.8		
	5	143	81.8	7.7	10.5	10.946	0.2
...I would put them in the stable	1	134	49.3	22.4	28.4		
	2	203	46.8	18.2	35.0		

	3	46	50.0	23.9	26.1		
	4	87	44.8	20.7	34.5		
	5	142	55.6	16.2	28.2	6.173	0.6
...I would lunge the horse	1	135	11.1	11.1	77.8		
	2	207	4.3	5.3	90.3		
	3	46	10.9	10.9	78.3		
	4	87	5.7	6.9	87.4		
	5	142	4.9	10.6	84.5	13.98	0.08
...I would walk the horse	1	134	84.3	7.5	8.2		
	2	205	73.2	12.7	14.1		
	3	46	76.1	8.7	15.2		
	4	87	70.1	13.8	16.1		
	5	144	71.5	16.0	12.5	10.933	0.2
Walking a horse aids movement of the gut	1	136	75.0	16.9	8.1		
	2	207	64.3	27.5	8.2		
	3	45	82.2	8.9	8.9		
	4	86	72.1	19.8	8.1		
	5	144	63.2	29.2	7.6	15.217	0.06
Walking a horse with colic eases its pain / discomfort	1	135	58.5	24.4	17.0		
	2	205	47.8	34.1	18.0		
	3	44	50.0	31.8	18.2		
	4	87	51.7	26.4	21.8		
	5	143	45.5	36.4	18.2	7.911	0.4
Walking a horse with colic prevents it rolling	1	136	77.9	7.4	14.7		
	2	206	67.5	15.0	17.5		
	3	45	75.6	11.1	13.3		
	4	87	60.9	25.3	13.8		
	5	144	66.7	17.4	16.0	16.32	0.04
Walking a horse reduces the chance of a twisted gut	1	135	56.3	26.7	17.0		
	2	204	38.7	38.2	23.0		
	3	45	60.0	26.7	13.3		
	4	87	50.6	31.0	18.4		
	5	144	42.4	41.0	16.7	17.33	0.03
My experience helps me identify when I need to call the vet if I suspect colic	1	135	92.6	6.7	0.7		
	2	208	80.3	7.2	12.5		
	3	46	91.3	6.5	2.2		
	4	86	79.1	11.6	9.3		
	5	144	80.6	13.9	5.6	30.94	<0.001
Knowledge of what's normal for my horse helps me identify what may be colic	1	136	99.3	0.7	0.0		
	2	208	97.1	0.5	2.4		
	3	46	100.0	0.0	0.0		

	4	86	97.7	2.3	0.0		
	5	144	98.6	1.4	0.0	13.986	*

†Owner typology groups; 1= Competing professional, 2= All round amateur, 3= Non-competing professional, 4= Friend/companion, 5= Competing amateurs.

*algorithm would not converge, small numbers in groups.

A/SA = combined responses for ‘agree’ and ‘somewhat agree’, N/Don’t know = combined responses for ‘neutral’ and ‘don’t know’, SD/D = combined responses for ‘somewhat disagree’ and ‘disagree’.
