# Supplementary material for: Could it be colic? Horse-owner decision making and practices in response to equine colic
Source: BMC Vet Res. 2014 Jul 7;10(Suppl 1):S1. doi: 10.1186/1746-6148-10-S1-S1 (PMC4122872; doi:10.1186/1746-6148-10-S1-S1)
Supplement: Scantlebury additional file 7 — Decisions surrounding veterinary treatment and owner typology group. [file 1746-6148-10-S1-S1-S7.PDF]

Additional file 7: Decisions surrounding veterinary treatment and owner typology

	Typology†	Total	A/SA (%)	Neutral (%)	SD/D (%)	Chi square	p
It is up to me to make decision to call a vet	1	136	97.1	1.5	1.5		
	2	205	97.1	2.0	1.0		
	3	46	93.5	2.2	4.3		
	4	86	95.3	1.2	3.5		
	5	143	97.2	1.4	1.4	3.876	<0.9 p >0.75
I would wait until I saw particular signs before I would call a vet	1	133	85.7	3.0	11.3		
	2	207	82.1	5.3	12.6		
	3	43	79.1	7.0	14.0		
	4	85	77.6	4.7	17.6		
	5	143	84.6	2.1	13.3	5.779	0.7
I would wait and see before calling a vet	1	135	42.2	16.3	41.5		
	2	205	37.6	9.8	52.7		
	3	42	42.9	4.8	52.4		
	4	96	34.4	8.3	46.9		
	5	142	46.5	11.3	42.3	10.91	0.2
How the horse responds to my actions has an influence on whether to call the vet	1	134	67.9	10.4	21.6		
	2	204	52.9	6.4	40.7		
	3	43	62.8	9.3	27.9		
	4	86	47.7	10.5	41.9		
	5	142	57.0	13.4	29.6	21.56	0.007
The cost of calling a vet is a barrier to me seeking assistance	1	136	11.8	8.8	79.4		
	2	205	9.3	3.9	86.8		
	3	45	8.9	4.4	86.7		
	4	86	3.5	7.0	89.5		
	5	143	14.0	4.2	81.8	12.68	0.1
Current financial status is a consideration before calling the vet	1	134	3.7	9.7	86.6		
	2	202	9.4	3.5	87.1		
	3	46	8.7	6.5	84.8		
	4	86	3.5	4.7	91.9		
	5	143	11.9	2.8	85.3	17.29	0.03
The financial worth of the animal is unimportant in decision to call the vet	1	136	76.5	1.5	22.1		
	2	206	83.0	0.5	16.5		
	3	46	78.3	0.0	21.7		
	4	87	88.5	0.0	11.5		
	5	143	80.4	1.4	18.2	8.732	<0.5 p >0.25
The use of the animal has an important influnce on whether I call the vet	1	134	8.2	5.2	86.6		
	2	205	5.9	1.0	93.2		

	3	46	0.0	2.2	97.8		
	4	86	0.0	1.2	98.8		
	5	141	6.4	1.4	92.2	23.97	*
If the horse is insured I am more likely to call the vet	1	135	18.5	11.9	69.6		
	2	203	12.3	3.0	84.7		
	3	46	13.0	2.2	84.8		
	4	86	14.0	5.8	80.2		
	5	142	21.1	2.8	76.1	21.45	0.006
If vet advised surgery I would consent	1	136	75.0	15.4	9.6		
	2	206	76.7	13.6	9.7		
	3	46	76.1	19.6	4.3		
	4	86	76.7	11.6	11.6		
	5	142	72.5	18.3	9.2	4.776	0.8
If the horse is insured I am more likely to consent to surgery	1	136	52.2	12.5	35.3		
	2	204	40.2	13.7	46.1		
	3	46	47.8	6.5	45.7		
	4	87	40.2	6.9	52.9		
	5	142	57.0	8.5	34.5	18.29	0.02
If the animal is young I am less likely to consent to surgery	1	136	7.4	14.7	77.9		
	2	206	4.4	11.7	84.0		
	3	46	2.2	8.7	89.1		
	4	87	4.6	11.5	83.9		
	5	141	7.1	15.6	77.3	6.458	0.6
If the horse is retired I am less likely to consent to surgery	1	136	44.1	12.5	43.4		
	2	205	25.4	13.2	61.5		
	3	46	30.4	8.7	60.9		
	4	87	21.8	8.0	70.1		
	5	142	45.8	10.6	43.7	32.7	<0.001
If the animal is well adapted for its use I am more likely to consent to surgery	1	135	34.8	17.0	48.1		
	2	205	16.1	15.6	68.3		
	3	46	19.6	19.6	60.9		
	4	87	13.8	14.9	71.3		
	5	141	21.3	22.7	56.0	25.78	0.001
If the horse is a pet I am more likely to consent to surgery	1	135	41.5	17.8	40.7		
	2	205	49.3	12.7	38.0		
	3	46	50.0	8.7	41.3		
	4	87	56.3	9.2	34.5		
	5	141	36.9	18.4	44.7	13.51	0.1
If the animal is financially valuable, I am more likely to consent to surgery	1	135	40.7	15.6	43.7		
	2	203	19.7	12.3	68.0		
	3	46	21.7	15.2	63.0		

	4	86	11.6	12.8	75.6		
	5	142	29.6	16.9	53.5	37.11	<0.001

†Owner typology groups; 1= Competing professional, 2= All round amateur, 3= Non-competing professional, 4= Friend/companion, 5= Competing amateurs.

A/SA = combined responses for ‘agree’ and ‘somewhat agree’, SD/D = combined responses for ‘somewhat disagree’ and ‘disagree’.
